# Identification of key biomarkers in Angelman syndrome by a multi-cohort analysis

**DOI:** 10.3389/fmed.2022.963883

**Published:** 2022-08-16

**Authors:** Yong Li, Junhua Shu, Ying Cheng, Xiaoqing Zhou, Tao Huang

**Affiliations:** ^1^Department of Pediatrics, Maternal and Child Health Hospital of Hubei Province, Tongji Medical College, Huazhong University of Science and Technology, Wuhan, China; ^2^Department of Pediatric Intensive Care Unit, Maternal and Child Health Hospital of Hubei Province, Tongji Medical College, Huazhong University of Science and Technology, Wuhan, China

**Keywords:** Angelman syndrome, diagnosis, biomarkers, RNA-seq, pediatrics

## Abstract

The Angelman Syndrome (AS) is an extreme neurodevelopmental disorder without effective treatments. While most patients with this disease can be diagnosed by genetic testing, there are still a handful of patients have an unrecognized genetic cause for their illness. Thus, novel approaches to clinical diagnosis and treatment are urgently needed. The aim of this study was to identify and characterize differentially expressed genes involved in AS and built potential diagnostic panel for AS by NGS sequencing. A multi-cohort analysis framework was used to analyze stem cell-derived neurons from AS patients in GSE160747 dataset. We identified three differentially expressed genes (ACTN1, ADAMTS2, SLC30A8) differentiates AS patients from controls. Moreover, we validated the expression patterns of these genes in GSE146640, GSE120225. Receiver operating characteristic (ROC) curves analysis demonstrated that these genes could function as potential diagnostic biomarkers [AUC = 1 (95% CI 1–1)]. This study may provide new approach for diagnosing patients with AS and helping to develop novel therapies in treating AS patients.

## Introduction

A rare neurodevelopment disorder called Angelman syndrome (AS) was first described in 1965 by Dr. Harry Angelman, an English pediatrician, who described three severely children with it (1). Symptoms of AS include delayed development, lack of speech, ataxia, and, occasionally, attacks. There is an estimated incidence of AS between 1/10,000 and 1/20,000 (2). Mutations of the maternal UBE3A (ubiquitin protein ligase E3A) gene cause AS in 8% of the cases (3, 4). UBE3A affects protein levels and function through ubiquitination. The UBE3A gene in neurons is imprinted: predominantly maternal alleles are expressed with little or no expression of paternal alleles (5). Unlike gene deletions, the UBE3A gene has a strong relationship with autism when duplicated or triple copied. AS is diagnosed if the patient meets the consensus clinical diagnosis criteria and/or demonstrates maternally inherited UBE3 allele expression or functional deficits. Since an estimated 90% of individuals with typical AS phenotypes can be identified through molecular genetics testing, The remaining 10% of AS patients have an as yet unknown genetic cause for the disease (6). Although rapid progress has been made in understanding the disease-causing process in AS, the gene-specific treatment is still limited (7).

Due to the rarity of AS in children, there is almost little research on the disease, let alone drugs. Finding new diagnostic modalities and potential therapeutic targets becomes more challenging. In this study, we analyzed samples from transcriptomics cohorts and identified novel biomarkers for AS diagnosis. Moreover, as a result of these differentially expressed genes, we can find potential therapeutic targets and gain a deeper understanding pathogenesis of AS.

## Materials and methods

### Data sources

We identified 3 prospective AS studies (GSE160747, GSE146640, GSE120225) that were potentially eligible for inclusion in the study. We downloaded datasets from the GEO database.^1^ We identified 3 datasets and divided them into two “training” and one “validation” dataset (Table 1).

**TABLE 1:** Demographic of the study in training and validation dataset.

GEO ID	Sample size	Sample type	Annotation
GSE160747	36	iPSC-derived neurons	Training
GSE146640	12	iPSC-derived neurons	Training
GSE120225	6	H9 human embryonic stem cell lines	Validation

### Gene expression data and statistics analyses

All transcriptomic data were normalized using the GC-Robust Multi-array Average. A log2 transformation was applied to all gene expression before analysis. To underestimates the between-trial variance, we used the DerSimonian-Laird random-effects combine gene expression effect sizes *via* Hedges’ g effect size. Moreover, based on gene effect size (ES > 1.3), and Fisher’s method false discovery rate (FDR < 0.9), we identified a subset of genes as the AS score.

### Creation of Angelman syndrome score

As a starting point, we ran a forward search using the MetaIntegrator R package to identify the parsimonious gene set best suited for diagnostic ability (8). A forward search begins with the gene that has the best discriminative ability, and then at each step adds the gene with the greatest weighted AUC. Once the weighted AUC reaches some threshold, no further additions can raise it further. Every time a new gene was added to the forward search, we determined the AS score in the following: Mean (upregulated genes)-Mean (downregulated genes).

## Results

### The three diagnostic biomarkers of Angelman syndrome in two training datasets

**FIGURE 1:**
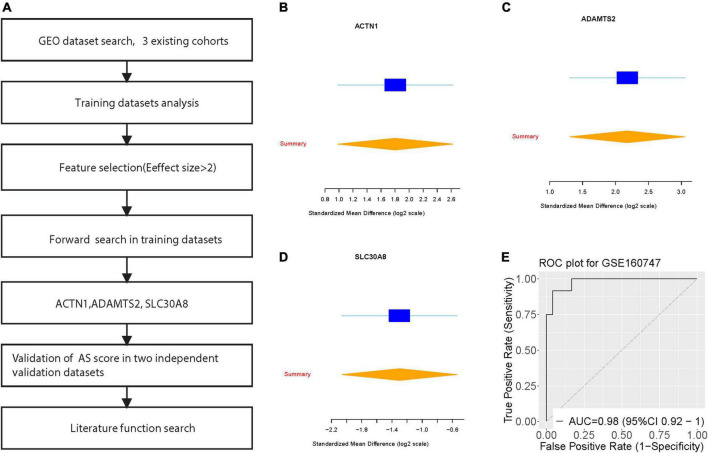
Discovery of three differentially expressed genes in diagnosis of Angelman syndrome. **(A)** Multi-cohort analysis workflow for identifying and validating the three differentially expressed genes. **(B–D)** The up-regulated gene (ACTN1) and down-regulated genes (ADAMTS2, and SLC30A8) from the forward searches in GSE160747. The x axis represents standardized mean difference between AS and dengue control. **(D,E)** ROC curves of patients with AS vs. controls in GSE160747.

We achieved a systematic search for data on transcriptome-wide expression between normal and AS tissue. According to the previously described method (ES > 1.3, FDR < 0.9), 12 genes were significantly upregulated, while 22 genes were significantly downregulated (Figure 1A). After forward search, we identified a set of 3 differentially expressed genes (ACTN1, ADAMTS2, SLC30A8) in AS/Normal that was optimized for diagnostic ability (Figures 1B–D). The AS signature (three diagnostic biomarkers) distinguished AS from normal subjects with a summary area under the curve (AUC) = 0.98 (95% CI 0.92–1) in the training dataset (Figure 1E).

### Validation of the three diagnostic biomarkers in two external datasets of Angelman syndrome

**FIGURE 2:**
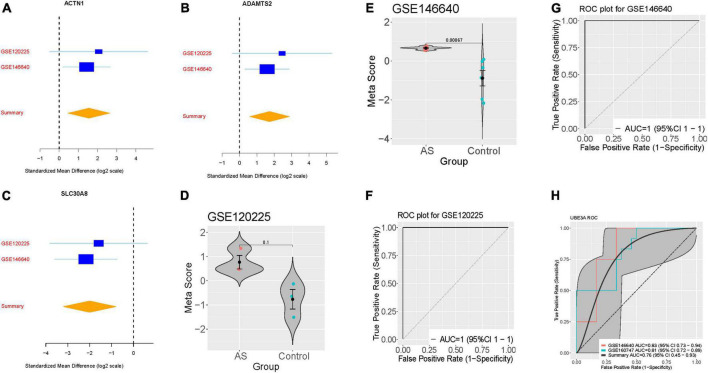
Validation of three differentially expressed genes diagnosis of AS. **(A–C)** The up-regulated gene (ACTN1) and down-regulated genes (ADAMTS2, and SLC30A8) searches in GSE120225 and GSE146640. The x axis represents standardized mean difference between AS and dengue control. **(D,E)** violin plot illustrating the meta-score of the three differentially expressed genes for differentiate AS from Control in GSE120225 and GSE146640. **(F,G)** ROC curves of patients with AS vs. controls in GSE120225 and GSE146640. **(H)** ROC curves of UBE3A diagnostic power in the training and validation dataset.

We further verified the AS signature in the two-validation set. For each dataset, we computed the effect size and meta-score by the previously described method (Figures 2A–C). Figures 2D,E violin plot showing the Meta-score of the 3-gene signature for separating AS from control group GSE120225 and GSE146640. The AUC = 1 (95% CI 1–1) differential AS from normal subjects by the AS signature in two validation datasets (Figures 2F,G). Meanwhile, we also compared with the well-known AS diagnostic marker UBE3A, which distinguished AS from normal subjects with a summary AUC = 0.76 (95% CI 0.45–0.93) (Figure 2H).

## Discussion

Families and individuals suffering from AS carry a heavy burden, due to the disease is so rare, treatment and medication are difficult to obtain. Currently, three treatment approaches for AS are in preclinical and clinical effect. The reintroduction of the UBE3A protein into the neurons through gene replacement therapies is one approach. It is also possible to “unsilence” paternal copies of the UBE3A gene. Thirdly, drugs that target proteins and effector mRNAs known to be affected in the pathophysiology of AS are being investigated (9).

In the current study, we identified three genes that are differentially expressed under UBE3A mutation in stem cell-derived neurons by mRNAs. As a result, we developed a model for AS scores using these three genes at the same time. Specifically, ACTN1, ADAMTS2, and SLC30A8 could serve as a biomarker to distinguish AS from normal patients. Alpha-actinin (ACTN) is an actin crosslinking protein. There are four varieties of ACTN, including two isoforms not found in muscles, ACTN1 and ACTN4.12. The non-muscle cytoskeletal protein, Alpha-actinin-1, is located at microfilament bundles and adherens-type junctions where it functions to bind actin to the cell membrane (10). To our knowledge, this is the first report of the ACTN1 have differential expression in in AS.

Studies suggest patients with missense mutation in the ACTN1 usually have an decreased number of large platelets and anisocytosis, but no *in vivo* changes to platelets (11). ADAMTS2 is an enzyme that clips a short chain of amino acids from the end of procollagens, which allows them to assemble into structural collagen molecules. The metalloproteinase ADAMTS-2 is responsible for processing fibrillar procollagen precursors to mature collagen molecules (12, 13). The Protein encoded by SLC30A8 (Solute Carrier Family 30 Member 8) is a zinc efflux transporter that impact the collection of zinc in intracellular vesicles (14). Interestingly, SNPs in SLC30A8 are extremely associated with Type 2 diabetes and gender-specific schizophrenia (15–17). It is likely that Zinc transporters or insulin secretion play a direct role in AS.

In spite of the extensive research, we have done on AS, there are still many limitations. Due to AS’s rarity, there are still a limited number of samples we can obtain. Meanwhile, the functions of these significantly expressed genes needs to be verified *in vitro* and *in vivo*. Developing and finding drugs that target these genes is also necessary.

## Data availability statement

The original contributions presented in this study are included in the article/supplementary material, further inquiries can be directed to the corresponding author.

## Ethics statement

The studies involving human participants were reviewed and approved by the Tongji Medical College. The patients/participants provided their written informed consent to participate in this study.

## Author contributions

TH and YL had the idea and launched the investigation. YL analyzed the results and drafted the manuscript. All authors contributed to the article and approved the submitted version.

## Footnotes

1
http://www.ncbi.nlm.nih.gov/geo/
